# Diversity of *Candidatus* Patescibacteria in Activated Sludge Revealed by a Size-‍Fractionation Approach

**DOI:** 10.1264/jsme2.ME22027

**Published:** 2022-06-08

**Authors:** Shuka Kagemasa, Kyohei Kuroda, Ryosuke Nakai, Yu-You Li, Kengo Kubota

**Affiliations:** 1 Department of Civil and Environmental Engineering, Tohoku University, 6–6–06, Aza-Aoba, Aramaki, Aoba-ku, Sendai, Miyagi 980–8579, Japan; 2 Bioproduction Research Institute, National Institute of Advanced Industrial Science and Technology (AIST), 2–17–2–1, Tsukisamu-Higashi, Toyohira-ku, Sapporo, Hokkaido 062–8517, Japan; 3 Department of Frontier Sciences for Advanced Environment, Tohoku University, 6–6–06, Aza-Aoba, Aramaki, Aoba-ku, Sendai, Miyagi 980–8579, Japan

**Keywords:** *Candidatus* Patescibacteria, candidate phyla radiation, size fractionation, activated sludge, diversity

## Abstract

Uncultivated members of *Candidatus* Patescibacteria are commonly found in activated sludge treating sewage and are widely distributed in wastewater treatment plants in different regions and countries. However, the phylogenetic diversity of *Ca.* Patescibacteria is difficult to examine because of their low relative abundance in the environment. Since *Ca.* Patescibacteria members have small cell sizes, we herein collected small microorganisms from activated sludge using a filtration-based size-fractionation approach (*i.e.*, 0.45–0.22‍ ‍μm and 0.22–0.1‍ ‍μm fractions). Fractionated samples were characterized using 16S rRNA gene amplicon and shotgun metagenomic sequence ana­lyses. The amplicon ana­lysis revealed that the relative abundance of *Ca.* Patescibacteria increased to 73.5% and 52.5% in the 0.45–0.22‍ ‍μm and 0.22–0.1‍ ‍μm fraction samples, respectively, from 5.8% in the unfractionated sample. The members recovered from the two size-fractionated samples included *Ca.* Saccharimonadia, *Ca.* Gracilibacteria, *Ca.* Paceibacteria, *Ca.* Microgenomatia, class-level uncultured lineage ABY1, *Ca.* Berkelbacteria, WS6 (*Ca.* Dojkabacteria), and WWE3, with *Ca.* Saccharimonadia being predominant in both fraction samples. The number of operational taxonomic units belonging to *Ca.* Patescibacteria was approximately 6-fold higher in the size-fractionated samples than in the unfractionated sample. The shotgun metagenomic ana­lysis of the 0.45–0.22‍ ‍μm fractioned sample enabled the reconstruction of 24 high-quality patescibacterial bins. The bins obtained were classified into diverse clades at the family and genus levels, some of which were rarely detected in previous activated sludge studies. Collectively, the present results suggest that the overall diversity of *Ca.* Patescibacteria inhabiting activated sludge is higher than previously expected.

Activated sludge processes have been applied to the treatment of various types of wastewater, including municipal and industrial wastewater. Microorganisms in activated sludge are key players in the removal of various pollutants (*e.g.*, organic matter, nitrogen, and phosphorus compounds). The microbial composition of activated sludge is affected by the type of wastewater, microorganisms in influent wastewater, the location of treatment plants, treatment processes, and operating conditions (*e.g.*, solid retention time) (Gonzalez-Martinez *et al.*, 2016; Xu *et al.*, 2018). Therefore, further knowledge of microbial phylogenetic diversity and abundance in various wastewater treatment processes is needed to obtain a more detailed understanding of activated sludge ecosystems. Although the morphology, diversity, physiology, and distribution of microorganisms involved in wastewater treatment are being documented and provided in databases, such as MiDAS (Nierychlo *et al.*, 2020), the functional roles of most microorganisms in activated sludge systems remain unclear because of the highly complex ecosystem as well as the presence of yet-to-be-cultured microorganisms (Chouari *et al.*, 2010; Sekiguchi *et al.*, 2015; Kindaichi *et al.*, 2016; Wang *et al.*, 2018).

Microorganisms belonging to *Candidatus* Patescibacteria (also known as candidate phyla radiation) are often found in wastewater treatment systems (Hu *et al.*, 2012; Zhang *et al.*, 2012; Ju and Zhang, 2015; Kindaichi *et al.*, 2016; Chin *et al.*, 2017; Li *et al.*, 2020; Hosokawa *et al.*, 2021; Martínez-Campos *et al.*, 2021; Singleton *et al.*, 2021). They also exist in activated sludge processes treating sewage, and major classes are *Candidatus* Saccharimonadia (TM7), *Candidatus* Paceibacteria (OD1, also known as *Candidatus* Parcubacteria), and *Candidatus* Gracilibacteria (GN02/BD1-5). We hereafter omit “*Candidatus*” to describe the patescibacterial phylogenetic groups for brevity. Patescibacteria is a taxonomic group with few culture representatives (*e.g.*, TM7x [He *et al.*, 2015]), and culture-independent approaches (*e.g.*, microautoradiography and fluorescence *in situ* hybridization [MAR-FISH] as well as metagenomics) have been adapted to elucidate their functions and roles in wastewater treatment processes. Saccharimonadia contributes to the degradation of sugar compounds in municipal wastewater treatment processes (Nielsen *et al.*, 2009; Kindaichi *et al.*, 2016). Saccharimonadia have also been suggested to be involved in ammoniacal nitrogen removal (Remmas *et al.*, 2017). Paceibacteria, Microgenomatia, and Gracilibacteria adapt to an anaerobic fermentation-based lifestyle and may play a role in hydrogen production and/or sulfur cycling (Wrighton *et al.*, 2012). In addition, Patescibacteria in an anammox reactor are expected to play roles in degrading the metabolic products of anammox bacteria and providing lactate and formate to other bacteria in order to maintain the anammox ecosystem (Hosokawa *et al.*, 2021).

Patescibacteria found in sludge treating wastewater are phylogenetically distinct among the samples analyzed; *e.g.*, Saccharimonadia found in sewage treatment plants in different countries and regions are not closely related (Kindaichi *et al.*, 2016). This implies that Patescibacteria involved in wastewater treatment are diverse, and their roles in activated sludge ecosystems may vary under different wastewater treatment operations. To obtain a more detailed understanding of Patescibacteria in activated sludge, a selective in-depth ana­lysis is crucial; however, it is hampered by the relatively low abundance of Patescibacteria in the complex microbial communities of activated sludge.

Patescibacteria is characterized by a small cell size (median diameter of 0.2‍ ‍μm, referred to as ultramicrobacteria) (Beam *et al.*, 2020). Studies on Patescibacteria in natural environments, such as seawater (Tully *et al.*, 2018), groundwater (Luef *et al.*, 2015; Ludington *et al.*, 2017; Probst *et al.*, 2017; Tian *et al.*, 2020; Chaudhari *et al.*, 2021), and freshwater lakes (Vigneron *et al.*, 2019) often involve performing the enrichment of small microorganisms by size fractionation as a pre-treatment process. This approach may be effective for the recovery of Patescibacteria from activated sludge. The present study attempted to enrich small microorganisms in sludge samples using a filtration-based size-fractionation approach and to elucidate the phylogenetic diversities of Patescibacteria in size-fractionated samples using 16S rRNA gene amplicon and metagenomic ana­lyses.

## Materials and Methods

### Sample collection and size fractionation

An activated sludge sample (designated as “unfractionated sludge”) was collected from the reaction tank of a wastewater treatment plant (WWTP) employing a conventional activated sludge process in Miyagi, Japan. The activated sludge process was operated at a solid retention time of 5.3‍ ‍d and hydraulic retention time of 5.7 h. Mixed liquor suspended solid and dissolved oxygen concentrations were 958 and 1.25‍ ‍mg L^–1^, respectively, and pH was 6.8 in the reaction tank at the time of sampling. Quality parameters for influent water to the reaction tank and effluent water from the final sedimentation tank were 84 and 6‍ ‍mg L^–1^ for biochemical oxygen demand, 26 and 27‍ ‍mg L^–1^ for total nitrogen, and 3.6 and 0.7‍ ‍mg L^–1^ for total phosphorus, respectively (average values of the sampling month). Regarding size fractionation, the activated sludge sample was centrifuged (7,000×*g* at 4°C for 5‍ ‍min). The activated sludge supernatant obtained was serially filtered through filters of decreasing pore sizes using a Stericup^®^ filter unit (filter area: 40‍ ‍cm^2^, Merck KGaA): 0.45‍ ‍μm (PVDF membrane, S2HVU02RE), 0.22‍ ‍μm (PVDF membrane, S2GVU02RE), and 0.1‍ ‍μm (polyethersulfone membrane, S2VPU02RE). The 0.22- and 0.1-μm filters were used as fractionated samples (designated as “0.45–0.22‍ ‍μm fraction” and “0.22–0.1‍ ‍μm fraction”, respectively). Samples were stored at –20°C until used.

### Fluorescence *in situ* hybridization (FISH)

The filtrate of the 0.45-μm filter was fixed with 2% paraformaldehyde at 4°C for 24 h. Subsequently, 30–50‍ ‍mL of the fixed sample was filtered through a polycarbonate membrane filter with a pore size of 0.2‍ ‍μm (diameter of 25‍ ‍mm; ADVANTEC). After drying, filters were dipped in 0.1% low-melting-point agarose (Invitrogen) in ultrapure water (w/v) for embedding (Pernthaler *et al.*, 2002). The filters were dried at room temperature and cut into 16 pieces. The TM7567 (5′-CCT ACG CAA CTC TTT ACG CC-3′) and TM7305 (5′-GTC CCA GTC TGG CTG ATC-3′) probes (Hugenholtz *et al.*, 2001) were used to detect Saccharimonadia. Both probes were labeled with Alexa Fluor 555 at the 5′-end. The TM7567 probe matched most of the 16S rRNA gene sequences of saccharimonadial operational taxonomic units (OTUs) obtained using the amplicon ana­lysis and bins obtained using the metagenomic ana­lysis (Fig. S1). Regarding hybridization, filters were incubated in hybridization buffer (20‍ ‍mM Tris-HCl [pH 7.5], 900‍ ‍mM NaCl, 1% blocking reagent [w/v] [Roche], and 0.01% sodium dodecyl sulfate [SDS, w/v]) containing 0.5‍ ‍μM of the probe at 46°C for 3 h. A specific formamide concentration was used for each probe, *i.e.*, 30% for TM7567 and 40% for TM7305. After the incubation, the filters were immersed in washing buffer (20‍ ‍mM Tris-HCl [pH 7.5], 0.01% SDS [w/v], 5‍ ‍mM EDTA, 103‍ ‍mM NaCl for TM7567, and 46‍ ‍mM NaCl for TM7305) at 48°C for 15‍ ‍min. The filters were dried and stained with 4′,6-diamidine-2′-phenylindole dihydrochloride (DAPI, 1‍ ‍μg‍ ‍mL^–1^; Merck KGaA). Microscopic observations were performed using Axio Imager 2 (Carl Zeiss) equipped with AxioCam HRm (Carl Zeiss).

### DNA extraction

DNA extraction from the unfractionated sludge sample was performed using the ISOIL for Beads Beating Kit (NIPPON GENE) by applying 0.1‍ ‍g wet sample. Half of the filter (approximately 20‍ ‍cm^2^) was used for fractionated samples. The filters were finely chopped into small pieces, immersed in 1.055‍ ‍mL of proteinase K solution (0.1‍ ‍mg mL^–1^ in 50‍ ‍mM Tris-HCl [pH 8.0], 100‍ ‍mM CaCl_2_, and 0.5% SDS), and incubated at 37°C for 3 h. Six hundred microliters of the solution was subjected to the ISOIL for Beads Beating Kit (DNA purification process only). DNA was eluted in 100‍ ‍μL TE buffer (10‍ ‍mM Tris-HCl and 1‍ ‍mM EDTA [pH 8.0]). DNA concentrations were measured using a Qubit^®^ 3 Fluorometer (Thermo Fisher Scientific) with the Qubit^®^ dsDNA HS Assay Kit (Thermo Fisher Scientific). The DNA yield of fractionated samples was calculated as follows: DNA concentrations were multiplied by the elution volume and divided by the number of filters used for DNA extraction.

### Amplicon ana­lysis targeting the 16S rRNA gene

Amplicon sequencing was performed to target the V3–V4 region of the 16S rRNA gene (Ni *et al.*, 2020). Primers without overhang sequences were used for the initial polymerase chain reaction (PCR) to minimize PCR bias (Berry *et al.*, 2011). The primer sets of 341F (5′-CCT AYG GGR BGC ASC AG-3′) and 806R-mix (a mixture of 806R [5′-GGA CTA CHV GGG THT CTA AT-3′] and 806R-P [5′-GGA CTA CCA GGG TAT CTA AG-3′] at a ratio of 30:1) were used (Matsubayashi *et al.*, 2017). The first round of PCR was performed using *TaKaRa Taq*^TM^ HS Low DNA (Takara Bio) and consisted of 25 amplification cycles (94°C for 5‍ ‍s, 50°C for 5‍ ‍s, and 68°C for 10 s), followed by a final extension step at 68°C for 7‍ ‍min. Forty amplification cycles were performed for the fractionated samples. PCR products were purified using Agencourt AMPure^®^ XP (Beckman Coulter). Primers with overhang sequences were used for the second round of PCR. The reaction was performed using *TaKaRa Ex Taq*^®^ Hot Start Version (Takara Bio) and consisted of a denaturing step at 94°C for 2‍ ‍min, followed by five amplification cycles (94°C for 30‍ ‍s, 50°C for 30‍ ‍s, and 72°C for 30 s), and a final extension step at 72°C for 5‍ ‍min. Purification was performed as previously described. The primers in Nextera^®^ XT Index Kit v2 Set A (Illumina) were used for the third round of PCR. The third round of PCR was performed using *TaKaRa Ex Taq*^®^ Hot Start Version, consisting of a denaturing step at 94°C for 2‍ ‍min, followed by eight amplification cycles (94°C for 30‍ ‍s, 55°C for 30‍ ‍s, and 72°C for 30 s), and a final extension step at 72°C for 5‍ ‍min. PCR products were verified for specific amplification using an Agilent Technology 2100 Bioanalyzer and Agilent DNA7500 Kit (Agilent Technology) and purified using Agencourt AMPure^®^ XP. The DNA concentration of the purified product was measured using a Qubit^®^ 3 Fluorometer with the Qubit^®^ dsDNA HS Assay Kit and adjusted to 4 nM by diluting it with 10‍ ‍mM Tris-HCl (pH 8.0). Sequencing of the products was conducted using an Illumina MiSeq sequencer (Illumina) with the MiSeq Reagent Kit v3 600-cycles (Illumina), following the manufacturer’s instructions.

Sequence data were initially subjected to a quality check (Trimmomatic ver. 0.39, SLIDINGWINDOW:6:20 MINLEN:200) (Bolger *et al.*, 2014). Passed sequence data were paired and checked for quality (read lengths: 300–500 bps, quality score: higher than 25) and chimeras (usearch61) using QIIME^TM^ ver. 1.8.0 software (Caporaso *et al.*, 2010). Single reads were removed using VSEARCH (Rognes *et al.*, 2016). OTUs were generated using a 97% sequence identity threshold. The SILVA 132 database (Quast *et al.*, 2013) was used for assignments. In this database, Paceibacteria are referred to as Parcubacteria. In the present study, we described Parcubacteria as Paceibacteria. OTUs assigned as “None” or “No blast hit” were reanalyzed with NCBI Blast (megablast, https://blast.ncbi.nlm.nih.gov/Blast.cgi) using the representative sequence of each OTU. As a result, approximately 90% of the OTUs assigned as “None” or “No blast hit” failed to match any species. Other OTUs hit viruses or plants with a low sequence identity. Therefore, OTUs assigned as “None” or “No blast hit” were removed from the ana­lysis. The number of sequence reads obtained were 26,078 from the unfractionated sludge sample, 25,035 from the 0.45–0.22‍ ‍μm fraction sample, and 7,383 from the 0.22–0.1‍ ‍μm fraction sample. To maintain the same sequencing depth, 7,000 randomly selected sequence reads were used for further data ana­lyses. The Goods coverage was 96.4–98.3%.

### Metagenomic ana­lysis

A metagenomic ana­lysis was performed using the 0.45–0.22‍ ‍μm fraction sample. Prior to library preparation, ethanol precipitation was conducted to concentrate DNA. The Nextera XT DNA Library Preparation Kit (Illumina) was used for library preparation. The prepared library was sequenced using a MiSeq sequencer with the MiSeq Reagent Kit v2 500-cycles (Illumina). Sequencing was performed twice using the same library.

Sequence data were trimmed based on the quality score using Trimmomatic ver. 0.39 (SLIDINGWINDOW:6:15, MINLEN:100 for data from the first sequencing run; SLIDINGWINDOW:6:30, MINLEN:100 for data from the second sequencing run). After co-assembly using the MEGAHIT ver. 1.2.9 (k-min 27, k-max 141, k-step 12) (Li *et al.*, 2015; 2016), contigs below 2,500‍ ‍bp were removed. Contigs were subsequently binned using the MetaBat2 ver. 2.15 (default settings) (Kang *et al.*, 2019), MaxBin 2 ver. 2.2.7 (markerset 40) (Wu *et al.*, 2014), Vamb ver. 3.0.2 (default setting) (Nissen *et al.*, 2021), and MyCC (MyCC_2017. ova) (Lin and Liao, 2016). All four sets of binned metagenomes were subsequently analyzed using DAS Tool ver. 1.1.2 (default settings) (Sieber *et al.*, 2018). Optimized non-redundant bins were dereplicated using dRep ver. 3.2.0 (Olm *et al.*, 2017) with the following parameter: -comp, 50. Quality checks of the bins were performed using CheckM ver. 1.0.7 with the marker set of cpr_43_markers.hmm for Patescibacteria genomes (Parks *et al.*, 2015). Bins with more than 50% completeness and contamination of 10% or less were selected and annotated using Prokka ver. 1.14.6 (Seemann, 2014). The Genome Taxonomy Database Toolkit (GTDB-Tk) ver. 1.5.1 (r202) (Chaumeil *et al.*, 2019) was used for the phylogenetic classification. The 16S rRNA gene sequences recovered from the bins were phylogenetically classified using the SILVA SSU Ref NR 99 132 database (Quast *et al.*, 2013). The BAM file was obtained using BBtool (bbmap.sh, ver. 38.18, input file1=trimmed forward read.fastq, input file2=trimmed reverse read.fastq, reference file=contig.fasta) and SAM tool ver. 1.4.1 (Li *et al.*, 2009). The median coverage of the bins was calculated using CoverM (-m relative abundance; https://github.com/wwood/CoverM) using the BAM file.

### Construction of phylogenetic trees

A phylogenetic tree was constructed based on the 16S rRNA gene sequences of Saccharimonadia, Gracilibacteria, Paceibacteria, Microgenomatia, and ABY1 obtained from amplicon and metagenomic ana­lyses. ARB software (http://www.arb-home.de/) (Ludwig *et al.*, 2004) and the SILVA SSU Ref NR 99 132 database (Quast *et al.*, 2013) were used. The phylogenetic tree was constructed using the neighbor-joining method implemented in ARB by extracting representative sequences of Saccharimonadia, Gracilibacteria, Paceibacteria, Microgenomatia, and ABY1 from the database. The 16S rRNA gene sequences obtained through amplicon (368 sequences) and metagenomic (18 sequences) ana­lyses were added to the constructed tree using the parsimony method.

A genome tree was constructed using the sequences of the obtained bins, Patescibacteria sequences recovered from WWTPs in Germany (Schneider *et al.*, 2021) and Denmark (Singleton *et al.*, 2021), and sequences from GTDB-Tk ver. 1.5.1 (r202). The tree was constructed using IQ-Tree 2 ver. 2.1.4-bet (Minh *et al.*, 2020) by the maximum-likelihood (ML) method (IQ-TREE multicore, <alignmentfile> bb 1000 -m LG+G4+FO+I). The model proposed by He *et al.* was referred to (He *et al.*, 2021).

### Deposition of DNA sequence data

The raw sequence data and sequence data of the metagenomic bins were deposited in the DDBJ Sequence Read Archive database (DRA013859).

## Results

### Microscopic observation of size-fractionated activated sludge

**Fig. 1.**
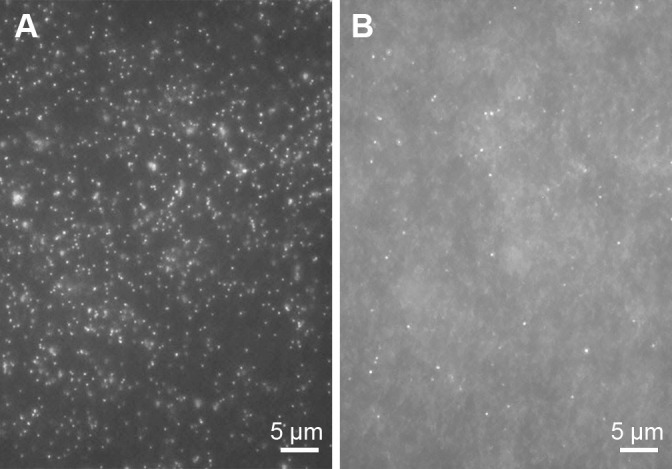
Detection of ultramicrobacteria (A) and *Candidatus* Saccharimonadia (B) in the size-fractionated sample (0.45–0.2‍ ‍μm fraction). DAPI-stained (A) and TM7567-derived fluorescence *in situ* hybridization (B) signals are shown in identical fields.

Microorganisms that passed through a pore size of 0.45‍ ‍μm and were trapped on the 0.2-μm filter were initially stained with DAPI, and we observed small coccoid cells (Fig. 1A). These results clearly indicated the presence of filterable ultramicrobacteria in activated sludge. Patescibacteria members, the target microorganisms of the present study, have a small cell size (Beam *et al.*, 2020). Therefore, we evaluated the sample by applying FISH to detect Saccharimonadia, a member of Patescibacteria known to exist in activated sludge. Two Saccharimonadia-specific probes, TM7305 and TM7567, were used, and the presence of Saccharimonadia in the size-fractionated sample was confirmed by detecting small coccoid-like cells with the TM7567 probe (Fig. 1B), but not with the TM7305 probe. *Candidatus* Saccharimonas aalborgensis from the activated sludge sample (Albertsen *et al.*, 2013) and TM7x from the human oral sample (He *et al.*, 2015; Bor *et al.*, 2018) were previously reported to have cell sizes of approximately 0.7 and 0.2–0.3‍ ‍μm, respectively. Similarly, our FISH experiment revealed that Saccharimonadia in the fractionated sample had a small cell size (expected to be between 0.2 and 0.45‍ ‍μm in diameter).

### DNA extraction efficiency from fractionated samples

Microorganisms in the supernatant after centrifuging the activated sludge sample were fractionated into the 0.45–0.22‍ ‍μm and 0.22–0.1‍ ‍μm fractions. The volume of the filtrate that passed through the 0.22- and 0.1-μm filters was approximately 3 L each. The concentrations of extracted DNA were 0.21‍ ‍ng μL^–1^ (0.45–0.22‍ ‍μm fraction sample) and 2.48‍ ‍ng μL^–1^ (0.22–0.1‍ ‍μm fraction sample). These DNA yields were equivalent to 0.04‍ ‍μg filter^–1^ (0.45–0.22‍ ‍μm fraction sample) and 0.50‍ ‍μg filter^–1^ (0.22–0.1‍ ‍μm fraction sample). Similar or higher DNA yields (average 1.19‍ ‍μg filter^–1^ [0.04–2.84‍ ‍μg filter^–1^, minimum to maximum] for 0.45–0.22‍ ‍μm fraction samples [*n*=6] and average 3.76‍ ‍μg filter^–1^ [0.22–15.10‍ ‍μg filter^–1^] for 0.22–0.1‍ ‍μm fraction samples [*n*=6]) were obtained from other activated sludge samples (these samples were not analyzed in the present study).

### Microbial community structures of fractionated activated sludge samples

**Fig. 2.**
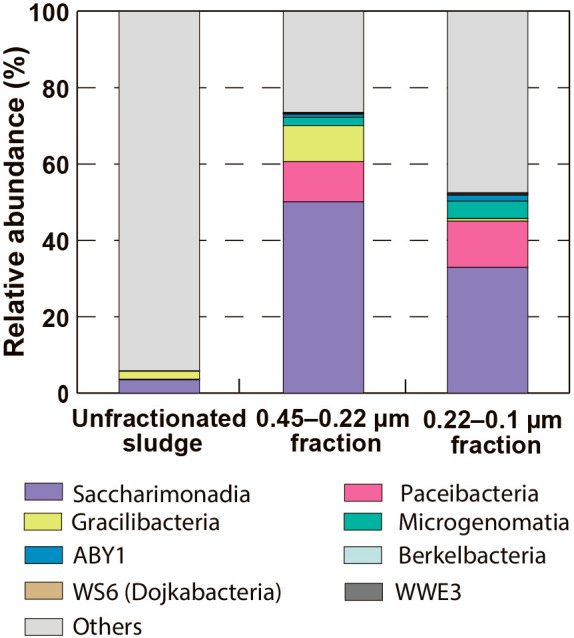
Community composition of *Candidatus* Patescibacteria. Phylogenetic groups other than *Ca.* Patescibacteria are included as “Others”.

The microbial community structures of the unfractionated sludge and the fractionated samples (*i.e.*, the 0.45–0.22‍ ‍μm and 0.22–0.1‍ ‍μm fractions) are shown in Table S1, and patescibacterial communities are highlighted in Fig. 2. The relative abundance of Patescibacteria was 73.5% for the 0.45–0.22‍ ‍μm fraction sample and 52.5% for the 0.22–0.1‍ ‍μm fraction sample. These values were higher than that of the unfractionated sludge sample of 5.8%. In the unfractionated sludge sample, five phylogenetic groups were detected in Patescibacteria: Saccharimonadia, Gracilibacteria, Paceibacteria, Microgenomatia, and ABY1, among which Saccharimonadia was dominant. In addition to these phylogenetic groups, Berkelbacteria, WS6 (Dojkabacteria), and WWE3 were detected in the fractionated samples. Saccharimonadia, Gracilibacteria, Paceibacteria, Microgenomatia, and ABY1 accounted for ≥1% of the relative abundance in the fractionated samples. Saccharimonadia was the dominant Patescibacteria, accounting for 59.7–68.1% (Fig. S2). Gracilibacteria showed a higher relative abundance in the 0.45–0.22‍ ‍μm fraction sample than in the 0.22–0.1‍ ‍μm fraction sample, whereas Paceibacteria, Microgenomatia, and ABY1 showed a higher relative abundance in the 0.22–0.1‍ ‍μm fraction sample than in the 0.45–0.22‍ ‍μm fraction sample. These results suggest that the degree of enrichment of Patescibacteria was influenced by the pore size of the filter used for size fractionation.

In addition to Patescibacteria, members of *Mollicutes* (Brown *et al.*, 2007), *Bdellovibrio* (Rendulic *et al.*,‍ ‍2004), *Polynucleobacter* (Hahn, 2003), and *Candidatus* Woesearchaeia (Castelle *et al.*, 2015) were detected in the fractionated samples. Some of these microorganisms have already been reported as ultramicrobacterial members that pass through 0.45- or 0.22-μm micropore filters (Nakai, 2020).

### Diversity of Patescibacteria in activated sludge

**Fig. 3.**
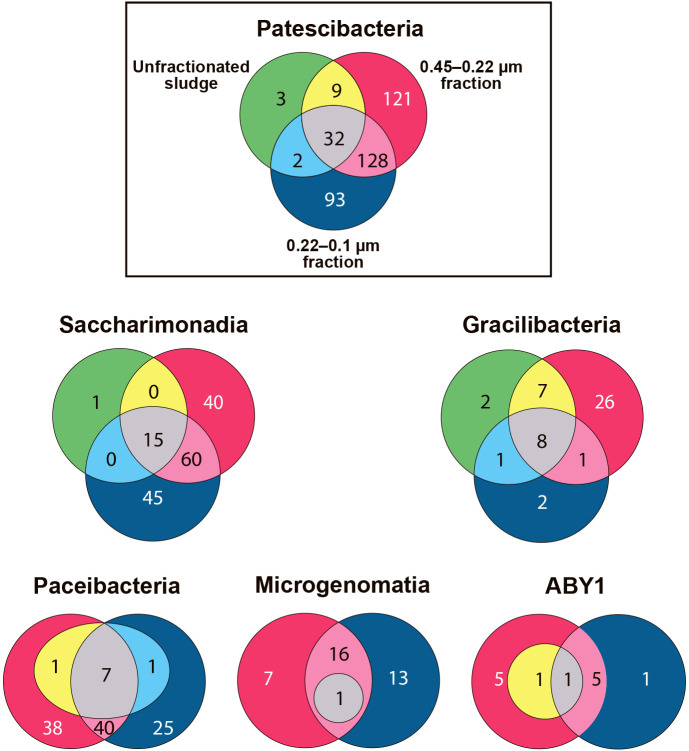
Number of OTUs overlapping among samples.

The numbers of OTUs and diversity indices of Patescibacteria were higher in the fractionated samples than in the unfractionated sludge sample. Table 1 shows the number of OTUs of Saccharimonadia, Gracilibacteria, Paceibacteria, Microgenomatia, and ABY1, and Fig. 3 depicts the overlap of OTUs belonging to the phylogenetic groups detected in each sample. Chao1, Shannon, and Simpson diversity indices are shown in Table S2. The number of OTUs belonging to the five phylogenetic groups was 46 for the unfractionated sludge sample, 279 for the 0.45–0.22‍ ‍μm fraction sample, and 242 for the 0.22–0.1‍ ‍μm fraction sample. The majority of OTUs detected in the unfractionated sludge‍ ‍sample were also found in the fractionated samples. Size fractionation enabled the retrieval of present but undetected Patescibacteria in samples.

A 16S rRNA gene-based phylogenetic tree was constructed for the five phylogenetic groups of Patescibacteria to identify the phylogenetic positions of the OTUs obtained in the present study (Fig. S1). OTUs were widely distributed among the phylogenetic groups, showing the high diversity of Patescibacteria in the activated sludge‍ ‍sample.‍ ‍The‍ ‍OTUs detected in the fractionated samples were phylogenetically close to those obtained from various environmental sources, such as bioreactors, soil, groundwater, and river water as well as the oral cavity. Most of the OTUs belonging to Paceibacteria, Microgenomatia, and ABY1 were phylogenetically related to those detected from size-fractionated groundwater and soil samples (pore size of 1.2–0.1‍ ‍μm) (Fig. S1). Most OTUs belonging to Saccharimonadia and Gracilibacteria were phylogenetically related to those detected in bioreactor sludge samples (note that some OTUs were phylogenetically related to those detected in environmental sources, such as soil and the oral cavity). OTU781, OTU958, and OTU1753 were phylogenetically close to Saccharimonadia and were detected in activated sludge from WWTPs in Denmark (Kong *et al.*, 2007; Albertsen *et al.*, 2013) and France (Chouari *et al.*, 2010). These three OTUs were only detected in the fractionated samples and were overlooked in the unfractionated sludge sample. This result indicates that various Saccharimonadia are present in activated sludge, but at a low abundance.

### Recovery of metagenome-assembled patescibacterial genomes

The 0.45–0.22‍ ‍μm fraction sample of activated sludge was used for further shotgun metagenomic sequencing to recover the genomes of Patescibacteria. We reconstructed 25 bins that passed the quality threshold (Table 2 and S3). Of the 25 bins, 24 belonged to Patescibacteria (one belonged to *Leptospiraceae*). Patescibacterial bins belonged to the taxonomic groups Paceibacteria, Saccharimonadia, ABY1, Gracilibacteria, and Microgenomatia. The expected genome size, completeness, and contamination of these bins are shown in Table 2. The bin-genome sizes recovered in the present study were similar to those in a previous study that predicted the genome size of Patescibacteria as 1.1±0.2‍ ‍Mbp (Tian *et al.*, 2020).

Eighteen 16S rRNA gene sequences were retrieved from the 15 bins of Patescibacteria (Table S4). The 16S rRNA gene sequences obtained by the metagenomic ana­lysis were phylogenetically close to the patescibacterial OTUs obtained by the amplicon ana­lysis (Table S5); however, these OTUs were not detected or were detected with low relative abundance (*i.e.*, 0.03–0.27%) in the unfractionated sludge sample. These results indicate that the size-fractionation approach is effective for recovering patescibacterial genomes with low abundance in activated sludge.

**Fig. 4.**
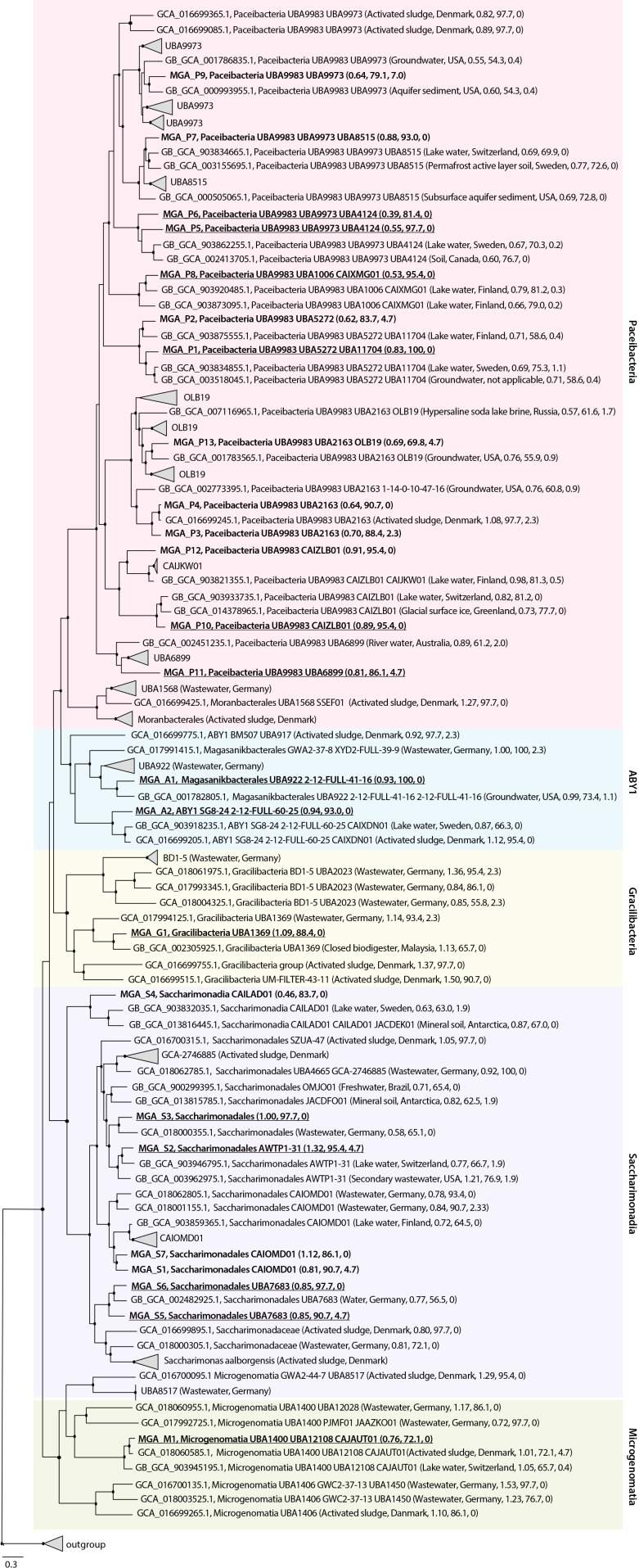
Genome-based maximum likelihood phylogenetic tree of *Candidatus* Patescibacteria. Bins obtained in this study are shown in red. 16S rRNA gene sequences were recovered from the underlined bins. Isolation source, geographic location, genome size (Mbp), completeness (%), and contamination (%) are shown in parentheses. Black circles at the nodes indicate bootstrap values of 95% or higher (1,000 replicates).

The construction of a genome tree revealed the phylogenetic positions of the bins obtained through the metagenomic ana­lysis (Fig. 4). The reconstructed patescibacterial bins belonged to various families and genera, some of which were rarely found in activated sludge samples. The bins of Paceibacteria belonged to six families: UBA6899, CAIZLB01, UBA2163, UBA5272, UBA1006, and UBA9973, while those of Saccharimonadia belonged to three families: UBA7683, CAIOMD01, and AWTP1-31; however, the bins of MGA_S3 and MGA_S4 were not identified at the family level. The two bins of ABY1 belonged to different families: UBA922 and 2-12-FULL-60-25. The bins of MGA_P4 (Paceibacteria), MGA_S3, MGA_S6 (Saccharimonadia), and MGA_M1 (Microgenomatia) were phylogenetically close to the genomes recovered from the WWTPs in Germany (Schneider *et al.*, 2021) and Denmark (Singleton *et al.*, 2021). In contrast, the bins of Gracilibacteria and ABY1 were not phylogenetically related to any of the genomes recovered from the WWTPs.

Our approach also recovered bins belonging to patescibacterial families or genera with a small number of genomes registered in the GTDB (as of April 2022). Eight bins belonged to families with ten or fewer registered genomes (UBA 7683, CAIOMD01, and AWTP1-31 in Saccharimonadia; UBA6899 and UBA5272 in Paceibacteria), and six belonged to the genus with five or fewer registered genomes (CAJAUT01 in Microgenomatia; 2-12-FULL-41-16 in ABY1; and UBA11704, CAIXMG01, and UBA4124 in Paceibacteria).

## Discussion

Although Patescibacteria is commonly present in activated sludge treating sewage, its ecophysiology and roles in sewage treatment processes remain unclear. The high microbial diversity and complexity of activated sludge ecosystems are bottlenecks for an in-depth ana­lysis of Patescibacteria. The cells of Patescibacteria are known to be significantly small and, thus, are classified as ultramicrobacteria (cell volume, <0.1‍ ‍μm^3^) (Luef *et al.*, 2015; Nakai, 2020). With this characteristic, physical size fractionation has been used to enrich ultramicrobacterial and patescibacterial members in natural environmental samples. Therefore, we applied a filtration-based size-fractionation approach to an activated sludge sample and successfully enriched small coccoid-like cells (Fig. 1A).

Most of the members enriched in the size-fractionated samples were Patescibacteria, *i.e.*, Saccharimonadia, Gracilibacteria, Paceibacteria, Microgenomatia, and ABY1, after the amplicon ana­lysis targeting the 16S rRNA gene (Fig. 2). The number of OTUs belonging to these phylogenetic groups increased (Table 1), and a high phylogenetic diversity of Patescibacteria in activated sludge was revealed (Fig. S1). The number of patescibacterial OTUs detected in activated sludge was more than 200, which was markedly higher than those found in natural environments, such as a groundwater sample (8 OTUs) (Chik *et al.*, 2020) and seawater sample (89 OTUs) (Suominen *et al.*, 2021), indicating that activated sludge harbors more Patescibacteria than natural environments. Water from various environmental sources, including households, industries, and nature, is collected and sent to WWTPs, suggesting that diverse Patescibacteria have the opportunity to migrate into the system. The stable physical conditions of sewage (*e.g.*, temperature and pH [Tian *et al.*, 2020]) may help Patescibacteria to survive in reaction tanks. In addition, if Patescibacteria adopts a parasitic/symbiotic lifestyle as previously suggested (He *et al.*, 2015; Moreira *et al.*, 2021; Yakimov *et al.*, 2022), biomass-rich activated sludge ecosystems provide more opportunities for contacting their hosts/partners than natural ecosystems, which is advantageous for the survival of Patescibacteria.

Patescibacteria have mainly been detected in anoxic or hypoxic environments (He *et al.*, 2021), and most are considered to be anaerobes based on their metabolic capacities predicted by genome ana­lyses (Castelle *et al.*, 2018). In contrast, some members of Patescibacteria are known to prefer aerobic conditions, and it has been reported that the phylogenetic groups detected and their proportions depend on the oxygen concentration in the ecosystem (Herrmann *et al.*, 2019; Chaudhari *et al.*, 2021). Saccharimonadia are often found in aerobic and anoxic environments, as shown in the present and previous studies (Albertsen *et al.*, 2013; Kindaichi *et al.*, 2016; Herrmann *et al.*, 2019; Chaudhari *et al.*, 2021). Paceibacteria and ABY1 exhibited different preferences for oxygen at the order/family level; *Candidatus* Kaiserbacteraceae in Paceibacteria showed a positive correlation with oxygen concentrations, while *Candidatus* Nomurabacteraceae/UBA9983 in Paceibacteria and *Candidatus* Magasanikibacterales in ABY1 showed negative correlations (Herrmann *et al.*, 2019). Therefore, each Patescibacteria member may find their niche in activated sludge flocs where an oxygen concentration gradient occurs.

Saccharimonadia are generally the dominant Patescibacteria in activated sludge treating sewage (Nielsen *et al.*, 2009; Kindaichi *et al.*, 2016) and have been clustered into at least six phylogenetic groups (G1-G6) (McLean *et al.*, 2020). Most of the OTUs detected in the present study belonged to group G1. G1 consists of sequences derived from various environmental sources, including bioreactors and mammalian host-associated (MHA) sources with strong human relevance. Many Saccharimonadia found in sludge treating wastewater, including *Ca.* Saccharimonas aalborgensis (Albertsen *et al.*, 2013), belong to this group. We found OTUs close to those detected in the activated sludge of WWTPs in other countries in the size-fractionated samples, but not in the unfractionated samples. Our in-depth ana­lysis revealed that these saccharimonadial OTUs were not specific to each WWTP, but were commonly present in activated sludge. In addition to the characteristics of municipal wastewater and operational conditions, the ecophysiological differences in these saccharimonadial OTUs may influence which OTUs become dominant in an activated sludge ecosystem. Therefore, further studies are needed to infer physiological and ecological characteristics from saccharimonadial genomic data.

The present study revealed the high diversity of Patescibacteria in activated sludge and demonstrated that size fractionation was effective for recovering patescibacterial genomes. Further studies to elucidate the ecophysiological characteristics of previously overlooked Patescibacteria will provide novel insights into activated sludge ecosystems. The filtration-based size-fractionation approach may be applied to other sludge samples, including anaerobic sludge. In addition, because a large amount of the patescibacterial biomass may be easily recovered using this technique, the cells obtained may also be used as an inoculum source to cultivate Patescibacteria members. The acquisition of additional Patescibacteria genomes and/or enrichment cultures through the size-fractionation approach will contribute to a more detailed understanding of the ecological characteristics of Patescibacteria in wastewater treatment processes.

## Citation

Kagemasa, S., Kuroda, K., Nakai, R., Li, Y.-Y., and Kubota, K. (2022) Diversity of *Candidatus* Patescibacteria in Activated Sludge Revealed by a Size-‍Fractionation Approach. *Microbes Environ ***37**: ME22027.

https://doi.org/10.1264/jsme2.ME22027

## Supplementary Material

Supplementary Material

## Figures and Tables

**Table 1.** Numbers of OTUs in five phylogenetic groups of *Candidatus* Patescibacteria

Samples	Saccharimonadia	Gracilibacteria	Paceibacteria	Microgenomatia	ABY1	Total
Unfractionated sludge	16	18	9	1	2	46
0.45–0.22‍ ‍μm fraction	115	42	86	24	12	279
0.22–0.1‍ ‍μm fraction	120	12	73	30	7	242

**Table 2.** Genome size, completeness, and contamination of patescibacterial bins obtained in the present study.

	Saccharimonadia	Gracilibacteria	Paceibacteria	Microgenomatia	ABY1
Number of bins	7	1	13	1	2
Average genome size (Mbp, Min–Max)	0.92 (0.46–1.32)	1.09	0.70 (0.39–0.91)	0.76	0.94 (0.93–0.94)
Average completeness (%, Min–Max)	91.7 (83.7–97.7)	88.4	88.9 (69.8–100)	72.1	96.5 (93.0–100)
Average contaminations (%, Min–Max)	2.0 (0–4.7)	0	1.8 (0–7.0)	0	0
