# Supplementary material for: Diversity of Candidatus Patescibacteria in Activated Sludge Revealed by a Size-‍Fractionation Approach
Source: Microbes Environ. 2022 Jun 8;37(2):ME22027. doi: 10.1264/jsme2.ME22027 (PMC9530733; doi:10.1264/jsme2.ME22027)
Supplement: Supplementary file 1 — Supplementary Material [file 37_22027_s1.pdf]

Supplemental material for

Diversity of *Candidatus* Patescibacteria in Activated Sludge Revealed by a Size-Fractionation Approach

Shuka KAGEMASA[1], Kyohei KURODA[2], Ryosuke NAKAI[2], Yu-You LI[1,3], Kengo KUBOTA[1,3*]

[1]Department of Civil and Environmental Engineering, Tohoku University, 6-6-06, Aza-Aoba, Aramaki, Aoba-ku, Sendai, Miyagi 980-8579, Japan; [2]Bioproduction Research Institute, National Institute of Advanced Industrial Science and Technology (AIST), 2-17-2-1, Tsukisamu-Higashi, Toyohira-ku, Sapporo, Hokkaido 062-8517, Japan; [3]Department of Frontier Sciences for Advanced Environment, Tohoku University, 6-6-06, Aza-Aoba, Aramaki, Aoba-ku, Sendai, Miyagi 980-8579, Japan

*Address correspondence to Kengo Kubota, E-mail; kengo.kubota.a7@tohoku.ac.jp

Tel & Fax: +81-22-795-5011

Table S1. Microbial community structures of the unfractionated sludge sample and the fractionated samples

Taxonomy		Rerative aboundance (%)		
Phylum/Domain	Class	Unfractionated sludge	0.45–0.22 μm fraction	0.22–0.1 μm fraction
Archaea	Micrarchaeia	N.D.*	0.03	N.D.
	Halobacteria	N.D.	0.01	0.03
	Methanobacteria	0.01	N.D.	N.D.
	Methanomicrobia	0.01	N.D.	N.D.
	Woesearchaeia	0.01	1.80	0.19
	Nitrososphaeria	N.D.	0.01	0.03
Acidobacteria	Acidobacteriia	0.20	N.D.	0.10
	Holophagae	0.61	0.01	0.06
	Subgroup 17	N.D.	N.D.	0.01
	Thermoanaerobaculia	0.04	N.D.	N.D.
Actinobacteria	Acidimicrobiia	0.33	0.04	0.06
	Actinobacteria	1.74	0.69	0.40
	Coriobacteriia	N.D.	0.03	N.D.
Bacteroidetes	Bacteroidia	24.47	3.57	4.67
	Ignavibacteria	2.30	0.06	0.71
Chlamydiae	Chlamydiae	N.D.	0.04	N.D.
Chloroflexi	Anaerolineae	1.94	N.D.	0.76
	Chloroflexia	0.04	N.D.	0.01
Cyanobacteria	Melainabacteria	N.D.	0.04	N.D.
	Sericytochromatia	0.26	N.D.	0.21
Deinococcus-Thermu	Deinococci	0.01	N.D.	N.D.
Dependentiae	Babeliae	0.09	0.41	0.10
Elusimicrobia	Elusimicrobia	0.01	0.11	0.01
	Endomicrobia	N.D.	0.04	0.01
	Lineage IIa	N.D.	0.10	N.D.
	Rs-M47	0.01	N.D.	N.D.
Epsilonbacteraeota	Campylobacteria	1.83	0.97	1.61
FCPU426	uncultured bacterium	N.D.	0.01	N.D.
Fibrobacteres	Fibrobacteria	N.D.	N.D.	0.06
Firmicutes	Bacilli	1.53	N.D.	0.04
	Clostridia	2.59	0.41	0.60
	Erysipelotrichia	0.29	0.06	0.01
	Negativicutes	0.14	0.01	N.D.
Fusobacteria	Fusobacteriia	0.29	0.04	0.46
Gemmatimonadetes	Gemmatimonadetes	0.03	N.D.	N.D.
Margulisbacteria	uncultured candidate division ZB3 bacterium	N.D.	N.D.	0.04
	uncultured organism	N.D.	0.30	0.06
Marinimicrobia (SAR406 clade); Ambiguous taxa		N.D.	N.D.	0.04
Patescibacteria	ABY1	0.07	0.80	1.56
	Berkelbacteria	N.D.	0.24	0.27
	Gracilibacteria	2.07	9.40	0.66
	Microgenomatia	0.03	2.16	4.57
	Parcubacteria	0.17	10.56	12.09
	Saccharimonadia	3.47	50.10	32.94
	WS6 (Dojkabacteria)	N.D.	0.11	0.10
	WWE3	N.D.	0.16	0.29
Planctomycetes	Planctomycetacia	0.01	N.D.	0.03
Proteobacteria	Alphaproteobacteria	6.07	7.89	9.04
	Deltaproteobacteria	6.93	3.43	3.61
	Gammaproteobacteria	40.86	3.87	13.23
Spirochaetes	Leptospirae	0.51	0.54	4.06
	Spirochaetia	0.46	0.63	5.86
	V2072-189E03	N.D.	0.03	0.11
Tenericutes	Mollicutes	0.03	1.16	1.14
Verrucomicrobia	Verrucomicrobiae	0.51	0.09	0.14
WOR-1	uncultured bacterium	N.D.	0.01	N.D.

* Not detected.

Table S2. Goods coverage, the number of OTUs, and diversity indices of *Candidatus* Patescibacteria

Samples	Goods coverage	OTUs	Chao1	Shannon	Simpson
Unfractionated sludge	94.6	46	69.1	3.15	0.726
0.45–0.22 μm fraction	97.8	290	409.4	4.4	0.763
0.22–0.1 μm fraction	98.8	255	268.9	5.67	0.942

Table S3. The quality and taxonomy of the bins obtained in this study

	Median coverage	Completeness (%)	Contamination (%)	Strain heterogeneity (%)	Genome size (Mbp)	N50 (scaffolds)	Taxonomy (database: GTDB-Tk ver. 1.5.1 (r202))
① MGA_P1	41.04	100	0	0	0.83	15594	Bacteria; Patescibacteria; Paceibacteria; UBA9983; UBA5272; UBA11704
② MGA_P2	9.09	83.7	4.7	0	0.62	8869	Bacteria; Patescibacteria; Paceibacteria; UBA9983; UBA5272
③ MGA_P3	12.69	88.4	2.3	100	0.70	9946	Bacteria; Patescibacteria; Paceibacteria; UBA9983; UBA2163
④ MGA_P4	11.90	90.7	0	0	0.64	36902	Bacteria; Patescibacteria; Paceibacteria; UBA9983; UBA2163
⑤ MGA_P5	16.11	97.7	0	0	0.55	65688	Bacteria; Patescibacteria; Paceibacteria; UBA9983; UBA9973; UBA4124
⑥ MGA_P6	11.04	81.4	0	0	0.39	11164	Bacteria; Patescibacteria; Paceibacteria; UBA9983; UBA9973
⑦ MGA_P7	48.34	93.0	0	0	0.88	17061	Bacteria; Patescibacteria; Paceibacteria; UBA9983; UBA9973; UBA8515
⑧ MGA_P8	13.31	95.4	0	0	0.53	12964	Bacteria; Patescibacteria; Paceibacteria; UBA9983; UBA1006; CAIXMG01
⑨ MGA_P9	7.15	79.1	7.0	0.0	0.64	9129	Bacteria; Patescibacteria; Paceibacteria; UBA9983; UBA9973; UBA9973
⑩MGA_P10	10.59	95.4	0	0	0.89	6991	Bacteria; Patescibacteria; Paceibacteria; UBA9983; CAIZLB01; CAIZLB01
⑪MGA_P11	7.89	86.1	4.7	0	0.81	8212	Bacteria; Patescibacteria; Paceibacteria; UBA9983; UBA6899; UBA6899
⑫MGA_P12	24.88	95.4	0	0	0.91	14518	Bacteria; Patescibacteria; Paceibacteria; UBA9983; CAIZLB01
⑬MGA_P13	15.22	69.8	4.7	100	0.69	9984	Bacteria; Patescibacteria; Paceibacteria; UBA9983; UBA2163; OLB19
⑭ MGA_S1	7.81	90.7	4.7	0	0.81	8639	Bacteria; Patescibacteria; Saccharimonadia; Saccharimonadales; CAIOMD01
⑮ MGA_S2	9.00	95.4	4.7	0	1.32	9484	Bacteria; Patescibacteria; Saccharimonadia; Saccharimonadales; AWTP1-31
⑯ MGA_S3	48.03	97.7	0	0	1.00	62819	Bacteria; Patescibacteria; Saccharimonadia; Saccharimonadales
⑰ MGA_S4	17.98	83.7	0	0	0.46	7011	Bacteria; Patescibacteria; Saccharimonadia; CAILAD01; CAILAD01
⑱ MGA_S5	6.93	90.7	4.7	0	0.85	7523	Bacteria; Patescibacteria; Saccharimonadia; Saccharimonadales; UBA7683
⑲ MGA_S6	28.27	97.7	0	0	0.87	74347	Bacteria; Patescibacteria; Saccharimonadia; Saccharimonadales; UBA7683; UBA7683
⑳ MGA_S7	28.27	86.1	0	0	1.12	20484	Bacteria; Patescibacteria; Saccharimonadia; Saccharimonadales; CAIOMD01
㉑ MGA_A1	13.02	100	0	0	0.93	153409	Bacteria; Patescibacteria; ABY1; Magasanikbacterales; UBA922; 2-12-FULL-41-16
㉒ MGA_A2	20.08	93.0	0	0	0.94	35899	Bacteria; Patescibacteria; ABY1; SG8-24; 2-12-FULL-60-25
㉓ MGA_G1	8.77	88.4	0	0	1.09	7844	Bacteria; Patescibacteria; GraciliBacteria; UBA1369; UBA1369
㉔ MGA_M1	7.18	72.1	0	0	0.76	7069	Bacteria; Patescibacteria; Microgenomatia; UBA1400; UBA12108; CAJAUT01
㉕MGA_Sp1	6.64	83.7	7.0	0	4.10	5394	Bacteria; Spirochaetota; Leptospirae; Leptospirales; Leptospiraceae; UBA2033

Table S4. Phylogenetic classification of the 16S rRNA gene sequences recovered by metagenomic analysis

Sequence ID*[1]	Length (bp)	Accession number of closest relative*[2]	Taxonomy (database: silva132)	Identity (%)
① MGA_P1_16S_1	1093	JX105604.1	Bacteria; Patescibacteria; Paceibacteria; uncultured bacterium	92.6
② MGA_P1_16S_2	1152	LN870984.1	Bacteria; Patescibacteria; Paceibacteria; Nomurabacteria; uncultured bacterium	93.8
③ MGA_P5_16S	994	JX105636.1	Bacteria; Patescibacteria; Paceibacteria; Nomurabacteria; uncultured bacterium	91.4
④ MGA_P6_16S	511	MFUC01000009.1	Bacteria; Patescibacteria; Paceibacteria; Nomurabacteria; Nomurabacteria bacterium RIFCSPHIGHO202FULL3815	85.7
⑤ MGA_P7_16S	1080	KC424740.1	Bacteria; Patescibacteria; Paceibacteria; Nomurabacteria; uncultured bacterium	96.2
⑥ MGA_P8_16S	1281	KP686931.1	Bacteria; Patescibacteria; Paceibacteria; Nomurabacteria; uncultured bacterium	95.3
⑦ MGA_P10_16S	1503	JX271992.1	Bacteria; Patescibacteria; Paceibacteria; Nomurabacteria; uncultured bacterium	83.9
⑧ MGA_P11_16S_1	1468	LN564834.1	Bacteria; Patescibacteria; Paceibacteria; Nomurabacteria; uncultured bacterium	93.1
⑨ MGA_P11_16S_2	457	HM243774.1	Bacteria; Patescibacteria; Paceibacteria; Moranbacteria; uncultured bacterium	89.8
⑩ MGA_S2_16S	423	CU925068.1	Bacteria; Patescibacteria; Saccharimonadia; Saccharimonadales; uncultured bacterium	95.8
⑪ MGA_S3_16S	1553	AB511000.1	Bacteria; Patescibacteria; Saccharimonadia; Saccharimonadales; uncultured bacterium	94.5
⑫ MGA_S5_16S_1	1537	KJ783099.1	Bacteria; Patescibacteria; Saccharimonadia; Saccharimonadales; uncultured bacterium	92.9
⑬ MGA_S5_16S_2	858	FPLM01001400.1	Bacteria; Patescibacteria; Saccharimonadia; Saccharimonadales; metagenome	97.6
⑭ MGA_S6_16S	428	GQ396872.1	Bacteria; Patescibacteria; Saccharimonadia; Saccharimonadales; uncultured bacterium	95.3
⑮ MGA_A1_16S	1490	MFQF01000003.1	Bacteria; Patescibacteria; ABY1; Magasanikbacteria; Magasanikbacteria bacterium RIFCSPHIGHO212FULL4116	92.5
⑯ MGA_A2_16S	1499	JF497854.1	Bacteria; Patescibacteria; ABY1; Candidatus Magasanikbacteria; uncultured bacterium	99.2
⑰ MGA_G1_16S	728	FJ189525.1	Bacteria; Patescibacteria; Gracilibacteria; Peregrinibacteria; uncultured bacterium	93.9
⑱ MGA_M1_16S	758	HM481340.1	Bacteria; Patescibacteria; Microgenomatia; Collierbacteria; uncultured bacterium	93.2
⑲ MGA_Sp1_16S	1516	FPLP01005991.1	Bacteria; Spirochaetes; Leptospirae; Leptospirales; Leptospiraceae; Leptospira; metagenome	99.5

*1 The ID was named as "bin’s name" + "_16S". When multiple 16S rRNA gene sequences were recovered from a single bin, consecutive numbers were added.

*2 The closest relatives were BLAST-searched using the SILVA_132_SSURef_NR99_tax database.

Table S5. Sequence identity between patescibacterial 16S rRNA gene sequences obtained from the metagenomic analysis and patescibacterial OTUs obtained from the amplicon analysis

*1 The relative abundance obtained by amplicon analysis.

Sequence ID	Identity (%)	OTU's ID	Relative abundance*[1] (%)	
			0.45-0.22 µm fraction	Unfractionated sludge
MGA_P1_16S_1	100	OTU 132	0.66	0.03
MGA_P1_16S_2	100	OTU 1169	0.50	N.D.*[2]
MGA_P5_16S	100	OTU 338	0.20	N.D.
MGA_P6_16S	100	OTU 1159	0.23	N.D.
MGA_P7_16S	100	OTU 1595	0.69	0.01
MGA_P8_16S	100	OTU 1377	0.43	N.D.
MGA_P10_16S	98.5	OTU 381	0.23	N.D.
MGA_P11_16S_1	100	OTU 1437	0.11	N.D.
MGA_P11_16S_2	99.3	OTU 833	0.29	N.D.
MGA_S2_16S	Not assigned.			
MGA_S3_16S	100	OTU 860	0.77	0.03
MGA_S5_16S_1	100	OTU 999	0.09	0.27
MGA_S5_16S_2	100	OTU 99	0.01	N.D.
	100	OTU 948	0.03	N.D.
	100	OTU 660	0.03	N.D.
MGA_S6_16S_2	Not assigned.			
MGA_A1_16S	83.1	OTU 1436	0.17	0.06
MGA_A2_16S	100	OTU 1436	0.17	0.06
MGA_G1_16S	97.8	OTU 897	3.67	0.26
MGA_M1_16S	Not assigned.			

*2 Not detected.

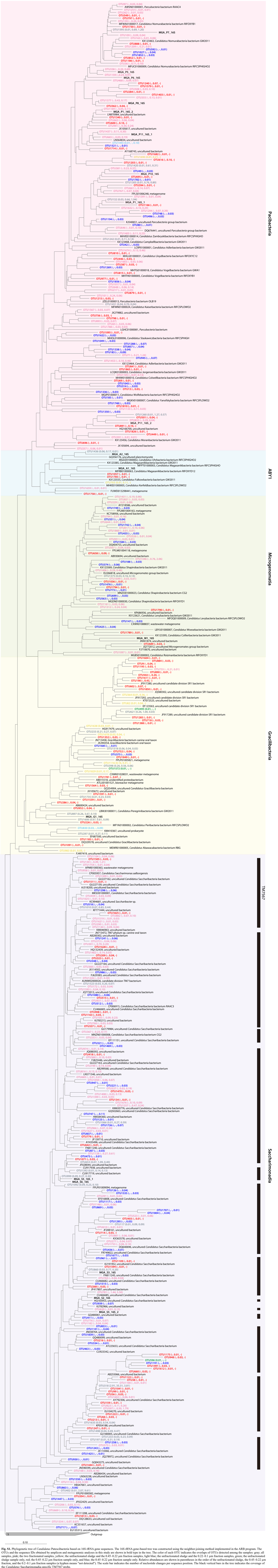

Fig. S1. Phylogenetic tree of *Candidatus* Patescibacteria based on 16S rRNA gene sequences. The 16S rRNA gene-based tree was constructed using the neighbor-joining method implemented in the ARB program. The OTUs and the sequence IDs obtained by amplicon and metagenomic analyses in this study are shown in bold type in the tree. The color of each OTU indicates the overlaps of OTUs detected among the samples: gray; all samples, pink; the two fractionated samples, yellow; the unfractionated sludge and the 0.45–0.22 μm fraction samples, light blue; the unfractionated sludge and the 0.22–0.1 μm fraction samples, green; the unfractionated sludge sample only, red; the 0.45–0.22 μm fraction sample only, and blue; the 0.45–0.22 μm fraction sample only. Relative abundances are shown in parentheses in the order of the unfractionated sludge, the 0.45–0.22 μm fraction, and the 0.2–0.1 μm fraction samples (a hyphen means "not detected"). The scale bar indicates the number of nucleotide changes per sequence position. The black vertical bars in the tree indicates the coverage of the *Candidatus* Saccharimonadia-specific TM7567 probe.

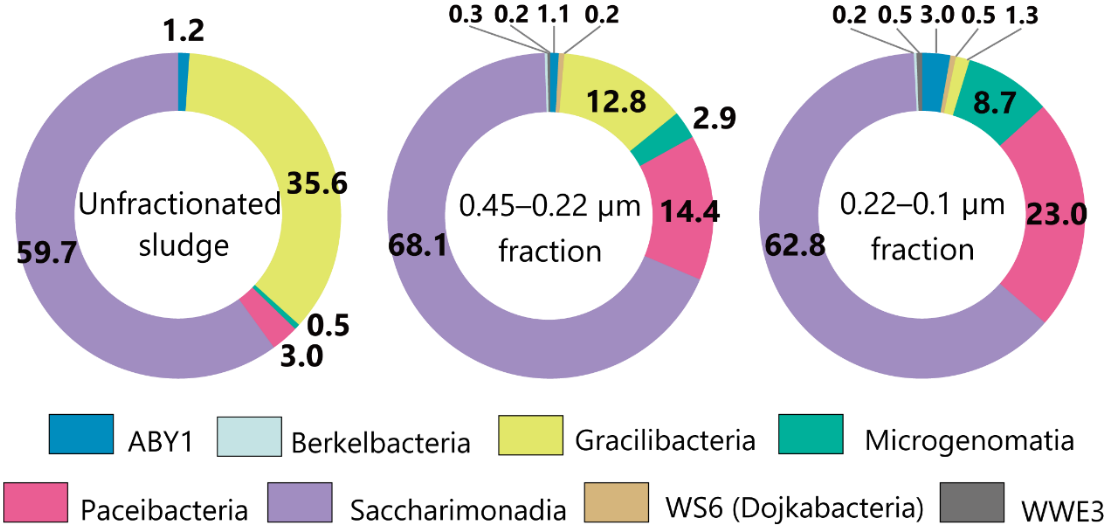

Fig. S2. Patescibacterial communities in the unfractionated sludge sample and the fractionated samples. The numbers in the figure indicate the relative abundance (%).
